# Evaluation of subcutaneous infiltration of autologous platelet-rich plasma on skin-wound healing in dogs

**DOI:** 10.1042/BSR20160503

**Published:** 2017-04-10

**Authors:** Haithem A. Farghali, Naglaa A. AbdElKader, Marwa S. Khattab, Huda O. AbuBakr

**Affiliations:** 1 Department of Surgery, Anesthesiology and Radiology, Faculty of Veterinary Medicine, Cairo University, Giza 12211, Egypt; 2 Department of Pathology, Faculty of Veterinary Medicine, Cairo University, Giza 12211, Egypt; 3 Department of Biochemistry and Nutrition, Faculty of Veterinary Medicine, Cairo University, Giza 12211, Egypt

**Keywords:** collagen1 A2, expression, immunohistochemistry, MMP, MDA, PRP

## Abstract

Platelet-rich plasma (PRP) is known to be rich in growth factors and cytokines, which are crucial to the healing process. This study investigate the effect of subcutaneous (S/C) infiltration of autologous PRP at the wound boundaries on wound epithelization and contraction. Five adult male mongrel dogs were used. Bilateral acute full thickness skin wounds (3 cm diameter) were created on the thorax symmetrically. Right side wounds were subcutaneously infiltrated with activated PRP at day 0 and then every week for three consecutive weeks. The left wound was left as control. Wound contraction and epithelization were clinically evaluated. Expression of collagen type I (COLI) A2, (COLIA2),histopathology and immunohistochemical (IHC) staining of COLI α1 (COLIA1) were performed on skin biopsies at first, second and third weeks. The catalase activity, malondialdehyde (MDA) concentration and matrix metalloproteinase (MMP) 9 (MMP-9) activity were assessed in wound fluid samples. All data were analysed statistically. The epithelization percent significantly increased in the PRP-treated wound at week 3. Collagen was well organized in the PRP-treated wounds compared with control wounds at week 3. The COLIA2 expression and intensity of COLIA1 significantly increased in PRP-treated wounds. MDA concentration was significantly decreased in PRP-treated wound at week 3. The catalase activity exhibited no difference between PRP treated and untreated wounds. The activity of MMP-9 reached its peak at the second week and was significantly high in the PRP-treated group. S/C infiltration of autologous PRP at the wound margins enhances the wound epithelization and reduces the scar tissue formation.

## Introduction

Skin wounds are the common and frequent purpose for research in veterinary practice. Healing of skin wounds can be challenging and more than one manner of repair may possibly be fruitful [1].

After skin injury, healing begins immediately. Blood clots seal the wound and provide a scaffold for cell migration [2]. Three to five days after injury; granulation tissue begins to replace the fibrin plug that fills the wound. Meanwhile, the re-epithelization starts to take place. The epidermal keratinocytes at the wound edge loosen their adhesion to each other and to the basal lamina, to cover the denuded area [2]**.** The greatest rate of collagen accumulation occurs between 1 and 2 weeks after injury rendering the wound its tensile strength. After 2–3 weeks, the wound begins to mature as collagen content and fibre orientation change [3,4]. Multiple regulators such as growth factors and cytokines, integrins, keratins, matrix metalloproteinases (MMPs), chemokines and extracellular macromolecules regulate these processes [2,5].

Cytokines and growth factors secreted during the wound healing process stimulate MMP. MMPs are a group of zinc-dependent extracellular proteinases expressed abundantly at wound edges [2,6]. They include stromelysin (1,3), collagenase 3, gelatinases (A, B) and membrane type 1 MMP. They are responsible for the degradation of extracellular matrix (ECM) proteins and release cryptic ECM molecules that regulate angiogenesis, secretion of chemotactic molecules and cell surface receptors to sustain keratinocyte migration [7,8].

During the inflammatory phase of wound healing, large amounts of reactive oxygen species (ROS) including superoxide radical (O_2_.^−^) and hydroxyl radical (OH) species, as well as non-radical oxygen derivatives that are capable of radical formation in cellular environment such as hydrogen peroxide (H_2_O_2_), hypochlorous acid (HOCl) and the thiol-targeting hypothiocynaous acid (HOSCN); are generated from inflammatory cells [9,10]. Subsequently, different strategies such as ROS-detoxifying enzymes: including superoxide dismutase, seleno-enzymes, GSH peroxidase and catalase were developed to maintain a redox homoeostasis protecting the proliferating and migrating cells in the wound tissue [11]. Besides that, the migrating neutrophils generate myloperoxidases catalyses the formation of the oxidants HOCl and HOSCN from H_2_O_2_ [10].

Platelet α-granules have many physiologically active substances to improve tissue regeneration, such as catecholamines, serotonin, calcium ions, ATP, fibrinogen, albumin, osteocalcin, osteonectin and various clotting factors [12]. The platelet released growth factors activate fibroblasts to produce proteoglycans, glycosaminoglycans and collagen, which eventually form the ECM [13]. The newly formed collagen is distributed in amounts that differ from that of the uninjured tissue [4]. The normal skin consists mainly of collagen type I (COLI) comprising approximately 80% of the dermis and 20% of collagen type III (COLIII) [4]. Collagens are proline-rich proteins that are fibrous with long, stiff and triple stranded helical structure [4]. In hypertrophic and immature scars, the percentage of COLIII may be as high as 33%, thus, most of the skin-healing therapies aim to increase the ratio of collagen I in the ECM of the wound [14]. The COLI triple helix is usually formed as a heterotrimer by two identical IA1 and IA2 chains. Symptoms associated with mutations in *COLIA2* gene, tend to be less severe than mutations in *COL1A1* gene since α-2 is less abundant reflecting the different role of these chains in matrix integrity. Entrez gene: collagen type I α-2 (*COL1A2* )[15].

Because platelets are a major source of healing factors within blood clots, therefore, concentrating platelets at the injured site could accelerate and optimize wound healing opening the door for the development of platelet-rich plasma (PRP) therapies. PRP is a platelet concentrate in a small plasma volume and its platelet-derived products have been used since the 1970s and they have been increasingly popular since the 1990s [16]. Autologous PRP is nowadays widely applied in different clinical applications to enhance the healing in orthopaedics, ophthalmology, dentistry and wound therapies [6,17].

PRP is a storage vehicle of active growth factors, such as platelet-derived growth factor (PDGF), platelet-derived angiogenesis factor (PDAF), platelet-derived epidermal growth factor (PDEGF), transforming growth factor (TGF)-β, platelet factor-4 (PF-4), insulin growth factor 1 (IGF 1), fibroblast growth factor (FGF) and endothelial growth factor (EGF), which are important in the regulation of healing process [6].

There are also many reports confirming the antibiotic effect of platelets by secreting antimicrobial peptides against different microorganisms and inhibiting bacterial growth besides the anti-inflammatory and analgesic effects possessed by it [18–20].

The aim of the present work was to evaluate the repair process of full thickness skin wounds after subcutaneous (S/C) infiltration of autologous PRP.

## Material and methods

### Animals

Five adult male mongrel dogs aged approximately 1–3 years and weighing 20–25 kg were used in the present study. After bilateral induction of full thickness skin wound at the thoracic region, the right sides of each dog were subcutaneously infiltrated with activated PRP and the left sides were left as untreated (control). The animals were kept in separate kennels under standard environmental conditions (23 ± 1°C, with 55 ± 5% humidity and a 12 h light/dark cycle). Free access to water and maintenance ration twice daily were available for all the dogs. The present study was approved by the Institutional Animal Use and Care Committee (IACUC). All surgeries were carried out under general anaesthesia, and all efforts were made to decrease animal suffering and number of used animals.

### Preparation of the autologous PRP

It was prepared using double spin method as a previously described protocol [21]. Briefly, Nine millilitres of each dogs’ whole blood was typically drawn from the left jugular vein on 1 ml of 3.8% sodium citrate solution in the 10 ml vacuum tube. PRP was isolated from the whole blood by soft spin at 250 × ***g*** for 10 min; three layers were obtained. The top and middle layers were collected directly and then directed to hard spin at 2000 × ***g*** for 10 min. After the second spin, the top two-third portion accepted as platelets-poor plasma was removed gently and then 1.5 ml PRP was obtained. PRP was activated using 20 mM CaCl_2_, then incubated at 37°C for 1 h. For recovery, the activated PRP was centrifuged at 3000 × ***g*** for 20 min. Then the prepared PRP from each dog was used as S/C infiltration at its induced skin wound margins.

### Creation of full thickness skin wound and subdermal application of the PRP

Under general injectable anaesthesia, each dog was pre-medicated with atropine sulphate (Atropine sulphate® 1%, Adwia Co., Egypt) at a dose of 0.1 mg/kg BW given subcutaneously and xylazine HCl (Xyla-Ject® 2%, Adwia Co., Egypt) at a dose of 1 mg/kg BW given intramuscularly. General anaesthesia was induced using ketamine HCl (Ketamine®, Sigmatec, Egypt) at a dose of 10 mg/kg BW and maintained by ketamine HCl [22]. Under complete aseptic conditions, bilateral circular full thickness skin wounds were created on the thorax symmetrically (3 cm diameter). Right side wounds were treated with S/C infiltration of 3 ml activated PRP. The left side wounds were left with the application of sodium fusidate cream (Fucidin®, Minapharm, Egypt) twice daily. Autologous activated PRP was applied at day 0 and then every week for three consecutive weeks.

### Wound fluid preparation

A standard wound fluid collection technique was carried out at the clinical site by a protocol previously described by [23]. Briefly, skin wounds were washed with sterile water prior to collecting wound fluid, followed by the application of an occlusive dressing over the wound. Exudates, which accumulated under the dressing after 30 min to 1 h, was recovered by washing with 1 ml of saline. The wound fluid samples were centrifuged at 14000 × ***g*** for 10 min. Protein content of all samples was measured as described by [24] Aliquots were made from samples and stored at –80°C until further analysis. Wound fluid samples were used for assessment of the catalase activity, lipid peroxidation and gelatin zymography.

### Clinical evaluation

For clinical evaluation, digital photographs were taken from wounds in the presence of ruler for measuring of wound contraction and epithelization at days 0, 7, 14 and 21 after injury. The wound contraction in both control and PRP-treated wounds was calculated according to the following equations:

$$\text{Percent of the wound size at the day }(\text{x})=\frac{\text{Wound size at the day }{\left(\text{x}\right)}^{\text{mm}2}}{\text{Wound size at the day }{\left(0\right)}^{\text{mm}2}}\times 100$$

100% of wound size at day (x) = percent of wound contraction

The wound epithelization was also calculated according to the following equation [25]:

$$\text{Percent of the epithelization }=\frac{\text{Size of epithelization area at the day }{\left(\text{x}\right)}^{\text{mm}2}}{\text{size of the wound at the day }{\left(\text{x}\right)}^{\text{mm}2}}\times 100$$

### Histopathological evaluation

The skin biopsies were taken from the centre and corner of each wound at 1, 2 and 3 weeks [25]. Part of the biopsies were fixed in 10% neutral formalin buffer and then processed by paraffin-embedding technique. Tissue sections 5 μm thick were made using microtome (Leica 2135). One slide from each sample at each period of time was stained with haematoxylin and eosin for analysis of tissue morphology and one other slide from each sample was stained with Masson’s trichrome for total collagen detection [26]. The tissue sections were examined by light microscopy and photographed using camera Olympus XC30 (Tokyo, Japan). The extent of re-epithelization, the presence of polymorphonuclear leucocytes (PMNL), fibroblasts and newly formed vessels, were assessed in a blinded manner according to a semiquantificative scoring system: 0 (absent), 1 (minimal), 2 (mild), 3 (moderate) and 4 (marked) [27]. Zero indicated no epithelization, absence of fibroblasts, PMNL or newly formed blood vessels, 1–increased thickness of cut edges of epithelium, presence of few fibroblasts, PMNL or newly formed blood vessels, 2–migration of epithelial cells, presence of moderate numbers of fibroblasts, PMNL or newly formed blood vessels, 3–epithelial bridging of the incision, the presence of many fibroblasts, PMNL or newly formed blood vessels. 4–complete regeneration of epithelium, presence of excessive numbers of PMNL, fibroblasts or newly formed blood vessels. In trichrome-stained slides, collagen abundance and collagen organization were assessed based on a scale of 0–3 for each characteristic. A score of 0 was given for samples with no collagen or disorganized collagen fibres. A score of 3 indicated abundant total collagen and well-organized fibres. Parallel wavy collagen fibres with consistent blue colour were considered to be more organized than collagen fibres that had variations of colour and breaks in parallel fibres or infiltration.

### Immunohistochemistry evaluation

Collagen type I α1 (COLIA1) was immunohistochemically stained in tissue sections. Paraffin-embedded tissue sections (5 μm thick) were mounted on positively charged slides (Citoglas®, Citotest Labware Manufacturing Co., Ltd, China). The tissue sections were then deparaffinized and rehydrated using xylene and descending concentration of ethanol [26]. The immunohistochemical (IHC) staining was carried out using the primary antibody against COLIA1 (Novus Biologicals, Europe) prepared in rabbits and avidin–biotin–peroxidase complex method (Dako, LSAB +system-HRP, North America, Inc.). All procedures were performed according to the manufacturer’s protocol. Colour development was carried out using DAB reagent and Mayer’s haematoxylin was used as a counterstain. Finally, the slides were dried, mounted with Canada balsam, covered and examined using the light microscope.

The intensity of colour on IHC stained slides was examined with a light microscope at 200× total magnification. The staining was scored on a scale from 0–4 based on the intensity of the resultant stain. A score of 0 indicated no staining, 1 indicated weak staining in few areas, 2 indicated weak staining in many areas, 3 indicated moderate staining in many areas and 4 indicated strong staining in many areas.

### Assessment of the catalase activity

Catalase reacted with a known quantity of H_2_O_2_ and the reaction is stopped after 1 min with catalase inhibitor. In the presence of peroxidase, the remaining H_2_O_2_ reacts with 3, 5-Dichloro-2-hydroxybenzene sulfonic acid and 4-aminophenazone to form a chromophore with a colour intensity inversely proportional to the amount of catalase in the sample. The absorbance was measured at 510 nm as described by [28].

### Assessment of lipid peroxidation

Malondialdehyde (MDA) concentration was used as the index of lipid peroxidation as described by [29]. MDA was determined by measuring the thiobarbituric acid reactive species. The absorbance of the resultant pink product was measured at 534 nm.

### Assessment of MMP activity

The activity of MMP-9 was detected in gelatin zymography by a method described by [30]. Briefly, wound fluid samples were separated by SDS/PAGE on 7.5% (w/v) gels, containing 1 mg/ml gelatin under non-reducing conditions. Then, it was washed twice for 15 min each in 2.5% (v/v) Triton X-100 and incubated in development buffer (0.05 M Tris/HCl, pH 8.8, 5 mM CaCl_2_, 0.02% NaN_3_) for 15 min to overnight incubation. Gels were stained with 0.1% Coomassie Brilliant Blue R250 in methanol:acetic acid:water (4.5:1:4.5, v/v/v).The zymograms gels were scanned in true colour and then analysed using commercially available software (myImageAnalysis Software; Thermoscientific™) after conserving to grey scale.

### Quantitative real-time PCR evaluation for COL1A2 expression

Total RNA isolation was performed in skin biopsies using QIAmp RNA mini kit (Qiagen, Hilden, Germany) according to the manufacturer’s manual. The concentration and purity of the total RNA samples were obtained by using a Nanodrop ND-1000 spectrophotometer. The isolated RNA was used for cDNA synthesis using reverse transcriptase (Fermentas, EU).

Real-time PCR (qPCR) was carried out using the reaction mixture of 1 μl cDNA, 0.5 mM of each primer (COL1A2 and GAPDH as an internal control), iQ SYBR Green Premix (Bio–Rad 170–880, U.S.A.) in a total volume of 20 μl. PCR amplification and analysis were achieved using Bio–Rad iCycler thermal cycler and the MyiQ realtime PCR detection system. All templates were amplified using the following Lightcycler protocol. The primer for COL1A2 was based on the sequence published in gene bank XM_005628344. One for *Canis lupus familiaris*; forward primer: CGGTCTCAGAGGCGAAATTG and the reverse one: CTCCCTTAGCACCAGGTTGA. The fast start polymerase was activated and cDNA denatured by pre-incubation for 5 min at 95°C, the template was amplified for 35 cycles of denaturation programmed for 45 s at 95°C, annealing of primers at 60°C programmed for 45 s and extension at 72°C programmed for 10 min. Fluorescent data were acquired during each extension phase. Each assay includes triplicate samples for each tested cDNAs and no-template negative control [31]. The Δ*C*_T_ value is calculated by the subtraction of the GAPDH *C*_T_ from each COL1A2 *C*_T_; in which *C*_T_ is the cycle number at which detectable signals are achieved. The Δ*C*_T_ value is calculated by subtraction of the control Δ*C*_T_ from each COL1A2 Δ*C*_T_. The expression relative to control is calculated using the equation 2^–ΔΔ*C*^_T_.

### Statistical analysis

Statistical analysis was performed by the statistical package SPSS, version 8.0 (SPSS Inc., Chicago, IL, U.S.A.). Statistical analysis of data was carried out using Student’s *t* test. Results were expressed as a mean ± S.E.M. *P* values less than 0.05 were considered significant.

## Results

### Clinical findings

**Figure 1:**
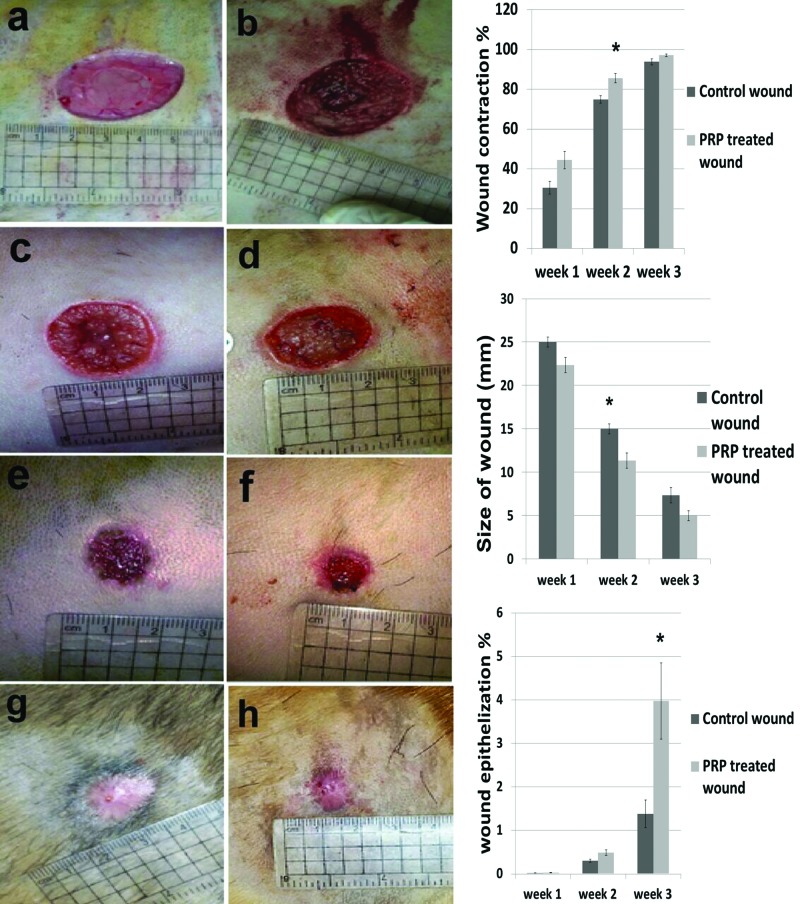
Clinical evaluation of wound healing in control and PRP treated wounds. Figure ( **1a)** photograph of control wound** and (b) PRP treated wound at 0 day, (c)** control wound and ( **d**) PRP treated **wound at 1 week** , **(e) control wound** and (f) PRP treated wound at 2 weeks, **(g) control wound** and (**h** ) PRP treated wound at 3 weeks. Graphs showing (**i** ) wound contration %, (**j**) Size of wound and (**k**) wound epithelization % in both control and PRP treated wounds.

The wounds induced at 0 day in both control and PRP treated groups were similar in size (Figure 1a, b). At 1 week, the PRP-treated wound showed smaller sized wounds in comparison with the control. Size of wound at week 1 was 25 mm in control (Figure 1c) compared with PRP-treated wound 22 mm (Figure 1d); week 2 control wound 15 mm (Figure 1e) and 10 mm in PRP -treated wound (Figure 1f) and week 3 control wound 5 mm (Figure 1g) in comparison with the PRP-treated wound 3 mm (Figure 1h). In this context, a significant size reduction (*P*<0.05) was found at week 2 (Figure 1i). There was increased wound contraction percentage (*P*<0.05) in the PRP-treated wound for all intervals with significant values at week 2 (Figure 1j). The re-epithelization rate percentages significantly increased in the PRP wound at week 3 (Figure 1k).

The actual measurements of wound size, contraction and re-epithelization were clarified by Table 1, which contained data that were presented as mean ± S.E.M.

**Table 1:** Clinical evaluations (size of wound, contraction percentage and the epithelization percentage) of control and PRP-treated wound

	Week 1	Week 2	Week 3
**I–Size of wound (mm)**			
Control	25 ± 0.58	15 ± 0.58	7.33 ± 0.58
PRP	22.33 ± 0.88	11.33 ± 0.88*	5 ± 0.88
**II–Contraction percentage %**			
Control	30.5 ± 3.18	74.93 ± 1.90	93.83 ± 1.48
PRP	44.41 ± 4.42	85.57 ± 2.29*	97.13 ± 0.64
**III–Epithelization percentage %**			
Control	0.02 ± 0.00	0.31 ± 0.035	1.38 ± 0.32
PRP	0.03 ± 0.00	0.49 ± 0.07	3.97 ± 0.87*

Data are presented as mean ± S.E.M.

*Significant difference: *P*<0.05. I- The size of wound in PRP treated wound compared to control wound. II-The contraction percentage % in PRP treated wound compared to control wound. III-The epithelization percentage % in PRP treated wound compared to control wound.

### Histopathological findings

**Figure 2:**
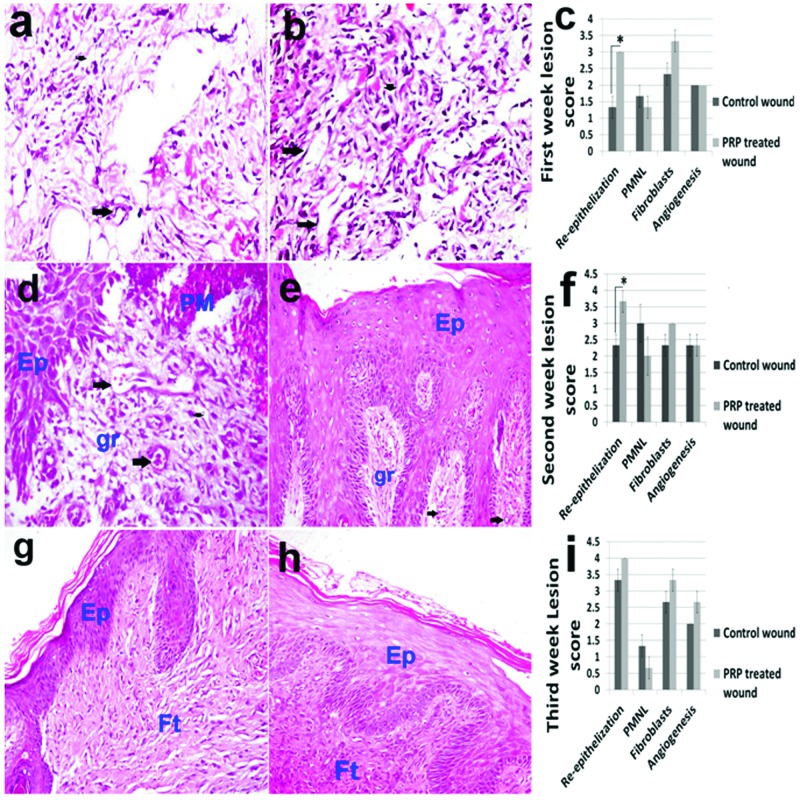
Histopathology and lesion score of wound healing in control and PRP treated skin wounds. Skin, dog. (**a**) Control wound and (**b**) PRP-treated wound at first week; fibroblasts infiltration (short arrow), angiogenesis (long arrow) (H&E ×400), (**c**) first week lesion score. (**d**) Control wound (H&E ×400) and (**e**) PRP-treated wound at second week (H&E ×200). Ep, epidermis; gr, granulation tissue; Pm, polymorphnuclear leucocytes; (**f**) Second week lesion score. (**g**) Control wound and (**h**) PRP-treated wound at third week; Ft, fibrous tissue (collagen) (H&E stain ×200); (**i**) Third week lesion score.

Polymorphonuclear cells were seen infiltrating the top of wound whereas marcrophages and migrating fibroblasts were observed at the base of the wound at the first week. The number of migrating fibroblasts in the PRP-treated wound was higher compared with the control wound. The epithelium at the wound edges began to proliferate in order to replace the lost epithelium site. Moreover, angiogenesis was noticed in both PRP-treated wound and control wound at the first week (Figure 2a,b). The re-epithelization recorded a significant increase in the PRP treated wound compared to control (Figure 2c).

At the second week of wound healing, the keratinized epithelium was restricted to the wound edges in control wound (Figure 2d) and had bridged the wound area in PRP-treated wound, however, the wound was still covered with a scab formed of necrotic debris and degenerated PMNL (Figure 2e). Interestingly, the re-epithelization recorded a significant increase in the skin treated with PRP compared with control at the second week (Figure 2f). The number of infiltrating PMNLs were increased in both control and PRP-treated wounds in the second week compared with the first week. However, PMNL was less observed in the PRP-treated wound compared with the control wound (Figure 2f). The granulation tissue was formed beneath the epithelium that was rich in fibroblasts and newly formed blood vessels. Using Masson’s trichrome stain, the collagen was not yet detected in the control wound and was poorly detected in the PRP-treated wound.

**Figure 3:**
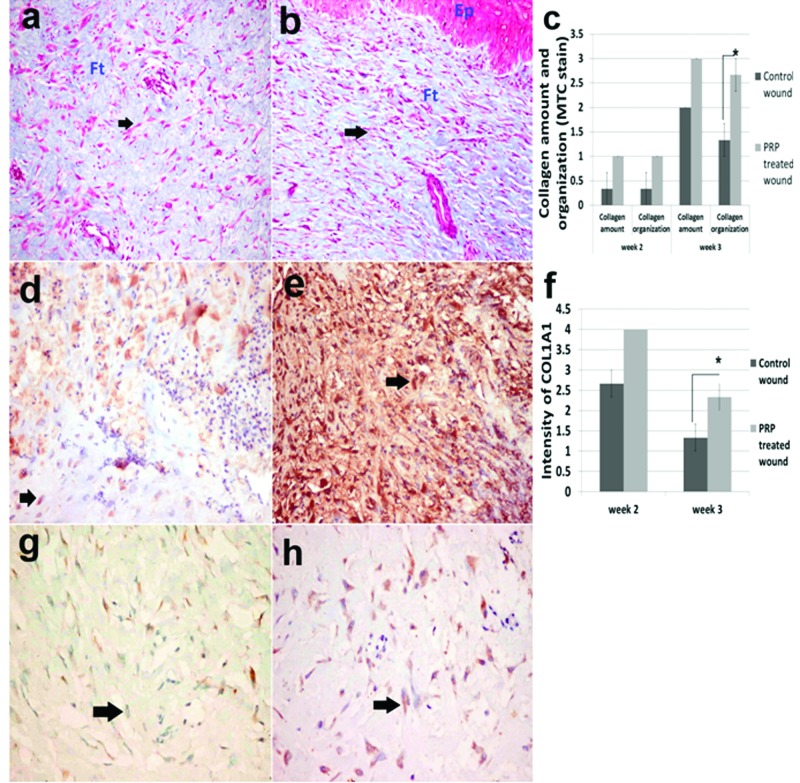
Collagen bundles and collagen IA1 staining in control and PRP treated skin wounds Skin, dog. (**a**) Control wound and (**b**) PRP-treated wound at third week. Fibroblasts indicated by arrows, EP, epidermis; Ft, fibrous tissue (collagen) (Masson’s trichrome ×200). (**c**) Lesion score for collagen amount and organization recorded in second and third week. (**d**) Brown coloured collagen IA1 (arrows) in the control wound and (**e**) in the PRP wound at the second week. (**f**) Evaluation of the intensity of collagen IA1 in IHC stained sections at the second and third week. (**g**) Collagen IA1 staining in the control wound and (**h**) in the PRP wound at third week (avidin–biotin–peroxidase complex method, Mayer’s haematoxylin counterstain ×400).

At the third week, the epithelium increased in thickness that was more observed in the wound treated with PRP (Figure 2g–i). The collagen was well stained with MTC at this time and the fibres were significantly well organized, having a parallel orientation and no separation between strands in the wound treated with PRP (Figure 3a–c). On the other hand, disorganized collagen found in random orientations and having inconsistent blue colour was seen in the control wound.

### IHC findings

**Figure 4:**
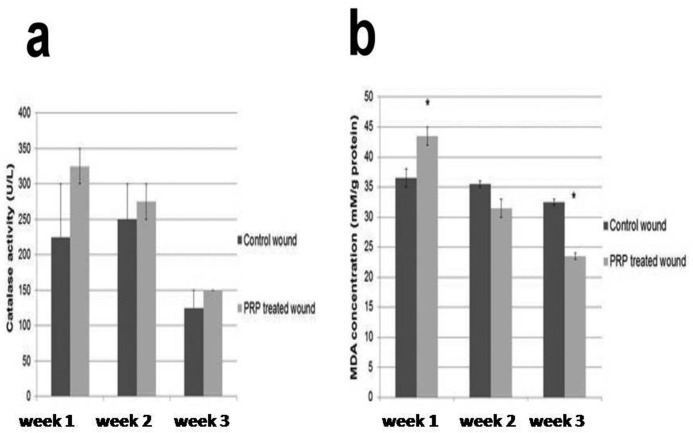
Evaluation of catalase activity and MDA concentration in control and PRP treated wounds. (**a**) catalase activity (U/L), (**b**) MDA concentration (mM/g protein) in control and PRP treated wounds.

The fibroblasts and granulation tissue stained brown for the COLIA1. The intensity of brown colour was significantly higher in the PRP skin wound in which strong staining in many areas was observed in the PRP wound at the second week compared with the control (Figure 3c,d). At the third week, the intensity of brown colour decreased and was only confined to the fibroblasts in both treated and untreated wounds However, the number of positively stained fibroblasts was higher in the PRP (Figure 3e,f and Figure 4h).

### Biochemical findings

There was no significant difference in catalase activity, between the treated and control wounds, at any time point (Figure 4a). In contrast, MDA was significantly increased in the PRP-treated wounds after the first week, but was decreased after 2 weeks (Figure 4b).

### MMP activity findings

**Figure 5:**
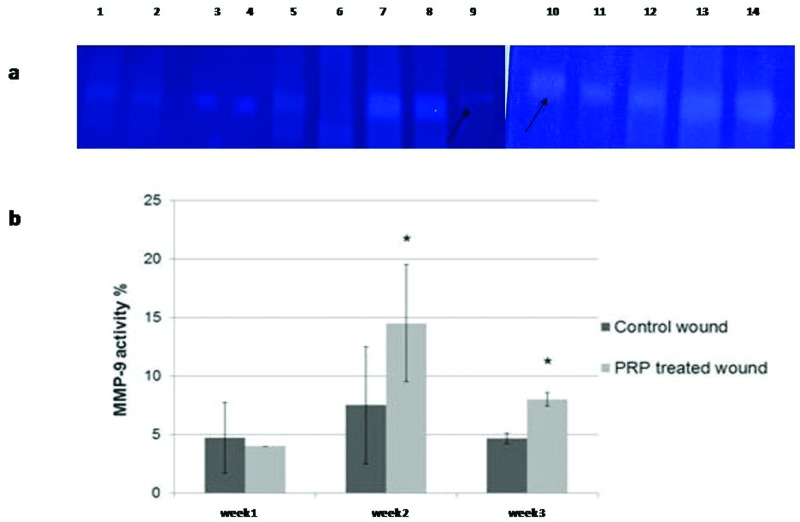
Activity of MMP-9 and gelatin zymography of enzyme activity in control and PRP-treated wounds (**a**) Lanes 1–2 = control week 1; lanes 3–4 = PRP-treated week 1; lanes 5–6 = control week 2; lanes 7–8 = PRP-treated week 2; lanes 11–12 = control week 3; lanes 13–14 = PRP-treated week 3. Positive controls shown in lanes 9 and 10 are from baby hamster kidney cells transfected with active MMP-9 (86 kDa) that are indicated by arrows and MMP-2 (66KDa) ‘not shown’. **(b)** Quantification of enzyme activity shown as % of bands intensity, the zymogram was quantified by MyImageAnalysis Software; Thermoscientific™.

The activity of MMP-9 was significantly increased in the PRP-treated wounds, compared with controls at weeks 2 and 3 (Figure 5a,b).

### Expression of COL1A2

**Figure 6:**
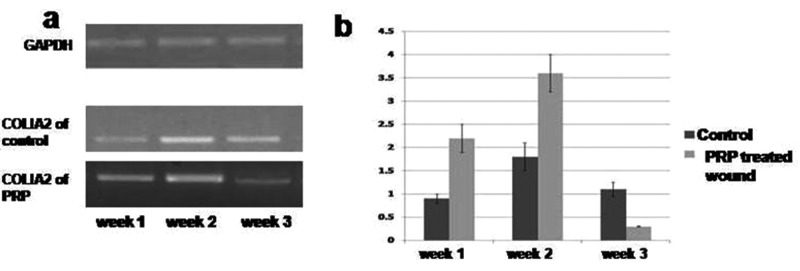
Quantitative RT-PCR of *collagen IA2* gene expression in PRP treated compared with control wound. (**a**) Electrophoretic mobility of quantitative RT-PCR products of *COLIA2* and *GAPDH* (internal control) genes on 2% agarose gel. (**b**) Evaluation of *collagen IA2* gene expression in PRP treated compared with control wound.

The expression of COLIA2 gradually increased in the PRP-treated skin wound by 0.4-fold change in comparison with the untreated wound. Then it’s expression significantly increased by 2-fold in the second week of PRP-treated wound followed by significant decreased in week 3 in comparison with the untreated wound. (Figure 6a,b).

## Discussion

In the present study, we prepared PRP from autologous blood, so it has the advantage of eliminating the risk of cross contamination, as well as the transmission of microbial diseases or immune reactions [32].

PRP was prepared by using the double spin method, in which blood cell layers were manually separated [21]. As well as, there are new technologies are used in PRP preparation by using ACP system (autologous conditioned plasma) [33], AutoloGel™ system for PRP diabetic ulcer treatment and commercially available kits [34]. Although these methods are recently available techniques; the classical centrifugal method was described as low cost preparation method, most accurate and rational choice for manual making [35,36].

After PRP preparation, it was activated exogenously by calcium chloride prior to S/C infiltration to initiate fibrin network formation easily after injection by solidifying the plasma and creating a fibrin clot or membrane in accordance with [21]. Beyond maintaining haemostasis, the fibrin clot then provides a matrix for the migration of tissue-forming cells and endothelial cells involved in angiogenesis and the remodelling of the clot into repair tissue**.** PRP is used in many clinical applications such as bone, cartilage, tendon and muscle lesions [36].

In the present study, the S/C infiltration of PRP was used in the wound margins rather than the topical application of the PRP gel because it stimulates the production of excessive inflammatory wound exudates that provide a moist wound environment; inhibiting wound contraction [37,38]. In accordance with the previous studies using PRP gel, there were no statistically significant differences found in the percentage of wound contraction, epithelization and healing process [39]. Our study is contradictory to these previous findings, although the number of PRP gel application to the wound should be daily more than PRP infiltration which is weekly.

The duration of the current study was only three weeks in accordance with previous studies [40]. The wound had healed rapidly and completely within 3 weeks of the first PRP treatment, without chronic effects or formation of exuberant tissue granulation and with minimum scarring.

In the present study, the activity of catalase in wound fluid was not significantly different between PRP treated and untreated skin all over the time of the study. However, its activity gradually decreased at the end of the study. Likewise, to the previous study, the level of expression of catalase mRNA was not changed during wound healing [41]. Catalase represents an attractive target to modulate the organelle’s antioxidative stress system in intact cells. Several approaches have already been used for this purpose; the most straightforward way is to incubate the cells with 3-amino-1,2,4-triazole (3-AT), a well characterized irreversible inhibitor of catalase. At non-cytotoxic dosages, catalase activity may decrease by ±70% [42]. Alternatively, genetically modified cells (e.g. from transgenic or knockout mice) [43], vector-driven expression systems or even cell-penetrating catalase. In this context, both reducing and increasing catalase activity may make cells more vulnerable to different types of oxidative stress [44]. Indeed, catalase null mice develop normally and do not display any gross physical or behavioural abnormalities. Nevertheless, tissues from these mice show a differential sensitivity to oxidant injury and exhibit a retarded rate in consuming extracellular H_2_O_2_ [43].

ROS-induced lipid peroxidation leads to the formation of several end products, including MDA. Increased MDA levels have been shown to be present in tissue homogenates and biological fluids, correlating with disease severity [45]. In the present study, there was a significant decrease (*P*<0.05) in MDA concentration in dogs treated with PRP in last week in comparison with the untreated wound in accordance with the recent studies [46]. Therefore, the results of oxidative biomarkers in the present study confirm that platelets have anti-inflammatory effects [18].

These results are in accordance with Wang and Nirmala [47] who revealed that the action of PRP can be complementary to tendon stem, progenitor cells in tendon injuries by supplying abundant growth factors contained platelets and fibrin matrix as a natural conductive scaffold to facilitate tissue healing. Moreover, Andia and Maffulli [48], have used PRP in the therapy of tendinopathy plantar fasciopathy and muscle injuries although they need to optimize protocols and obtain more high quality clinical data for PRP treatment recommendations.

The PRP infiltrations were found to enhance both the re-epithelization and wound contraction along the study period. Histopathologically, the thickness of epithelium was higher in the PRP-treated wound from the first week than the control and was more noticeable at second week in the present study. PRP is known to be rich in growth factors such as TGF-β, which decreases the proliferation of basal keratinocyte and induces the differentiation of suprabasal cell, therefore, stimulating the epidermal regeneration associated with cutaneous wound healing [25,49,50]. This increase in re-epithelization could be attributed to gelatinase A (MMP-2) and gelatinase B (MMP-9) expression in wound fluid since a significant increase of MMP-9 at the second and third weeks were recorded in the present study. Gill and Parks [49] supported the importance of MMP in cell migration and re-epithelization after the activation of both progelatinases during cutaneous wound healing in microdissected rat wound tissues by using zymographical analysis.

During re-epithelialization, there were increased activities of MMP-9 but not of MMP-2. Correspondingly, Sato et al. [51] observed high levels of mature gelatin B in the migrating epithelial sheet during human skin wound healing. Therefore, gelatinases may serve as indicators of the progression of the wound healing process.

In the present work, the skin tensile strength for PRP-treated and control wound was evaluated by the expression, amount and arrangement of collagen. The collagen is a major extracellular ECM constituent for maintenance of skin tensile strength and elasticity. The increase in wound tensile strength that takes place during the fibroblastic phase corresponds to the increasing levels of collagen within the wound [14,50].

In the present study, the evaluation included both COLIA2 and COLIA1 that form COLI, which accounts for 80% of the skin collagen with 4:1 in ratio with COLIII [4]. In the present study, COLIA2 expression in PRP-treated wound is significantly higher than its expression in untreated wound at the week 1 and 2 indicating that PRP was able to increase the expression of COLIA2. Moreover, the intensity of COLIA1 in immunohistochemically stained sections was higher in the skin treated with PRP compared with control at the second week. This result could be attributed to the presence of TGFs and other cytokines in PRP that initiate the conversion of fibroblasts into myofibroblasts, which are responsible for wound contraction and deposition of additional matrix proteins [5].

Later, COLIA2 expression and stain intensity of COLIA1 in the present study significantly decreased in last week in both treated and untreated wounds, although it was still higher in PRP-treated wound. The fibroblast migration and deposition of newly synthesized type 1 procollagen as well as other matrix molecules, replacing the provisional network composed of fibrin and fibronectin, begins on the third day of wounding and lasts for approximately 2 weeks thereafter [52]. Synthesis and breakdown of collagen as well as ECM remodelling, takes place continuously and both tend to equilibrate to a steady state approximately 3 weeks after injury [3].

In the early stages of granulation tissue, the collagen bundles are thin, randomly organized and contain many fibroblasts, whereas at the later stage the collagen bundles are dense, tightly packed and organized [53]. In the present study, the collagen formed dense, parallel, wavy bundles in the skin wound treated with PRP and was not yet organized in the control at the third week, which means that the PRP accelerated the maturation of granulation tissue. Similarly, [54] found that wounds treated with PRP exhibited more rapid epithelial differentiation and enhanced organization of dermal collagen. This positive effect of PRP could be attributed to the increase of MMP-9 since it was reported previously that MMP-9 is crucial to the assembly of collagen fibre primarily types I and III collagens [7].

## Conclusion

From the present data, it could be concluded that application of autologous PRP using S/C infiltration route at the wound margins showed significant enhancement of wound re-epithelization and reduced scar formation. This route of administration showed less number of applications (once per week), anti-inflammatory effects and potent antimicrobial action in addition to the action of PRP as a biological wound healing enhancer.

## Abbreviations

COLI: collagen type I
COLIA1: collagen type I α1
COLIII: collagen type III
COL1A2: collagen type I α2
DAB: diaminobenzidine
ECM: extracellular matrix
GSH: reduced glutathione
IHC: immunohistochemical
MDA: malondialdehyde
MMP: matrix metalloproteinase
MTC: Masson's trichrome
PMNL: polymorphonuclear leucocyte
PRP: platelet-rich plasma
qPCR: quantitive polymerase chain reaction
ROS: reactive oxygen species
Rt-PCR: real time polymerase chain reaction
S/C: subcutaneous
TGF: transforming growth factor
